# Dietary curcumin nanoparticles promoted the performance, antioxidant activity, and humoral immunity, and modulated the hepatic and intestinal histology of Nile tilapia fingerlings

**DOI:** 10.1007/s10695-022-01066-4

**Published:** 2022-04-05

**Authors:** Mohsen Abdel-Tawwab, El-Sayed Hemdan Eissa, Wesam A. Tawfik, Heba E. Abd Elnabi, Saadea Saadony, Walaa K. Bazina, Ragaa A. Ahmed

**Affiliations:** 1 Department of Fish Biology and Ecology, Central Laboratory for Aquaculture Research, Agriculture Research Center, Abbassa, Abo-Hammad, Sharqia, Egypt; 2 Mariculture Research Center, Arish University, El-Arish, Egypt; 3 Holding Co. for Biological Products and Vaccines, Giza, Egypt; 4 Naqaa Nanotechnology Network NNN, Giza, Egypt; 5 Department of Fish Resources and Aquaculture, Faculty of Environmental Agricultural Sciences, Arish University, El-Arish, Egypt; 6 Department of Animal Production and Fish Resources, Faculty of Agriculture, Suez Canal University, Ismailia, Egypt; 7 National Institute of Oceanography and Fisheries, Cairo, Egypt; 8 Department of Aquaculture, Faculty of Fish and Fisheries Technology, Aswan University, Aswan, Egypt

**Keywords:** Nile tilapia, Curcumin nanoparticles, Growth performance, Digestive enzymes, Antioxidants and immunity biomarkers

## Abstract

The current study investigated the effects of dietary curcumin nanoparticles (C-NPs) on the performance, hemato-biochemical profile, digestive enzymes activities, antioxidant status, humoral immunity, and liver and intestinal histology of Nile tilapia (*Oreochromis niloticus*). Fish (4.3 ± 0.5 g) were fed with diets enriched with 0.0 (control), 15, 30, 45, and 60 mg C-NPs/kg diet up to apparent satiety thrice a day for 60 days. The growth-stimulating effects of dietary C-NPs were significantly observed in terms of final weight, weight gain %, specific growth rate, and feed intake. Compared with the control group, serum amylase, lipase, and proteases activities of Nile tilapia significantly (*P* < 0.05) increased alongside the increase in dietary levels of C-NPs in a dose-dependent manner. The counts of red blood cells and white blood cells as well as hemoglobin and hematocrit levels of Nile tilapia fed with 30–60 mg C-NPs/kg diet were statistically (*P* < 0.05) higher than fish in the control group with no significant differences among them (*P* > 0.05). Moreover, lymphocytes and monocytes significantly (*P* > 0.05) increased; meanwhile neutrophils significantly (*P* > 0.05) decreased as C-NPs levels in diets increased. In a similar trend, antioxidant (malondialdehyde, superoxide dismutase, catalase, and glutathione peroxidase) and humoral immunity (lysozyme and total immunoglobulin) biomarkers were significantly higher in C-NPs-fed fish. Liver histology showed improvements in the cell architecture of fish fed with C-NPs containing diets up to 45 mg/kg diet. Compared with the control diet, feeding Nile tilapia with C-NPs diets resulted in a higher villi length/width and absorption area. According to the regression curves, the current study recommends using the dietary C-NP with optimum values of 45–55 mg/kg diet to improve the performance, digestive enzymes, antioxidant activities, and immunity response of Nile tilapia.

## Introduction

The most farmed fish in Egypt is Nile tilapia (*Oreochromis niloticus*), and it accounts for 51.7% of fish farming and with 357,639 tons produced in 2017; thus, Egypt ranked first tilapia producer in Africa and the third largest producer in the world (FAO 2018). Nile tilapia is omnivorous, accepts artificial feeding in intensive production systems, and exhibits rapid growth with high market value (El-Sayed 2019). Currently, the aquaculture industry is enhanced by optimizing the use of phytogenic natural compounds, which have been widely applied in aquafeeds to enhance the performance, antioxidant activity, and innate immunity of several fish species (Dawood et al. 2018; Dawood et al. 2020; Alagawany et al. 2021).

Turmeric (*Curcuma longa* L.), an important medicinal plant is a tropical, perennial, and rhizomatous herb belonging to Zingiberaceae family (Prasad et al. 2014). Curcumin is a hydrophobic and polyphenolic compound and one of the main active compounds of turmeric extract, which serves as a spice and dye in food preparation (Moghadamtousi et al. 2014; Hewlings and Kalman 2017). Furthermore, dietary turmeric or curcumin shows antimicrobial, antioxidant, anti-inflammatory, immune-modulatory, appetite-increasing, and gastro-protective effects on fish health (Sahu et al. 2008; Mahfouz 2015; Abdel-Tawwab and Abbass 2017; Abdelkhalek et al. 2021; Alagawany et al. 2021). Enis Yonar et al. (2019) reported that dietary curcumin substantially enhanced the growth and feed utilization of rainbow trout (*Oncorhynchus mykiss*). In addition, Mahmoud et al. (2017) reported that dietary curcumin significantly improved the growth indices and the protein efficiency ratio in Nile tilapia. Similarly, crucian carp (*Carassius auratus*) fed with dietary curcumin displayed enhanced growth indices and feed utilization (Jiang et al. 2016). Mohamed et al. (2020) detected that dietary fortification with curcumin quadratically improved growth indices and feed utilization of Nile tilapia.

With all benefits of curcumin, there are certain limitations to its utilization such as low water solubility, unstable chemical structure, rapid metabolism, and poor absorption in the body (Siviero et al. 2015; Hewlings and Kalman 2017). Curcumin in nanoparticle form exhibits better dispersion in aqueous media and better absorption than traditional bulk curcumin (Kurita and Makino 2013; Hani and Shivakumar 2014; Ghalandarlaki et al. 2014; Moniruzzaman and Min 2020). Numerous studies proved the positive effects of nano-sized feed additives to improve the performance and welfare status of several fish species (Korni and Khalil 2017; Abdel-Tawwab et al. 2018a; Abdel-Tawwab et al. 2019; Younus et al. 2020; Moghadam et al. 2021; Abdel-Tawwab et al. 2021). The nano-sized materials have ability to remain in the blood stream for long period hence enhance their bioavailability (Nair et al. 2016). It is hypothesized that dietary curcumin nanoparticles (C-NPs) can promote the fish performance and welfare status. Therefore, the current study investigated the effects of dietary supplementation of C-NPs on the performance, hemato-biochemical parameters, digestive enzymes activity, antioxidant status, and humoral immunity of Nile tilapia. Hepatic histology and intestinal histomorphometry were also investigated.

## Materials and methods

### C-NPs preparation

A syringe pump containing antisolvent was used to prepared C-NPs in accordance with the method of Kakran et al. (2012) with slight modifications using dichloromethane as an organic solvent (Carvalho et al. 2015). The original curcumin solution was prepared in dichloromethane (5 mg/mL), filled into the 20-mL syringe, and injected (10 mL/min) into the deionized water (antisolvent) at ratio of 1:12 under magnetic stirring (1000 × *g*) for 2 h. The formed NPs were filtered and vacuum dried. The C-NP dimension was determined using a Zeta sizer (Malvern Instruments, Zeta sizer nano series Nano-s, UK). The mean diameter of the particles was 82.7 ± 11.1 nm.

### Experimental diets and fish rearing

All ingredients and proximate chemical composition of a control diet containing 30% crude protein (CP) were shown in Table 1. The control diet was enriched with C-NPs at levels of 0.0 (control), 15, 30, 45, and 60 mg/kg diet. Then, the C-NPs were suspended in 100-mL distilled water and added to diets ingredients by uniform spraying, mixed well for 30 min, and pelleted (1–2 mm diameter). The prepared diets were stored in plastic bags at −4°C for further use.

**Table 1:** Ingredients and approximate chemical composition (% on dry matter basis) of the control diet

Ingredient	%	Chemical composition	%
Fish meal (72% CP)	11	Dry matter	91.5
Soybean meal (45% CP)	36	Crude protein	31.3
Wheat bran	20	Ether extract	6.1
Yellow corn	6	Total ash	7.3
Rice bran	20	Crude fiber	6.6
Fish oil	1.5		
Soybean oil	1.5		
Dicalcium phosphate	1		
Vitamins mixture^1^	1		
Minerals mixture^2^	1		
Carboxymethyl cellulose	1		
Total	100		

^1^Vitamin premix (per kg of premix): thiamine, 2.5 g; riboflavin, 2.5 g; pyridoxine, 2.0 g; inositol, 100.0 g; biotin, 0.3 g; pantothenic acid, 100.0 g; folic acid, 0.75 g; para-aminobenzoic acid, 2.5 g; choline, 200.0 g; nicotinic acid, 10.0 g; cyanocobalamine, 0.005 g; a-tocopherol acetate, 20.1 g; menadione, 2.0 g; retinol palmitate, 100,000 IU; cholecalciferol, 500,000 IU

^2^Mineral premix (g/kg of premix): CaHPO_4_.2H_2_O, 727.2; MgCO_4_.7H_2_O, 127.5; KCl 50.0; NaCl, 60.0; FeC_6_H_5_O_7_.3H_2_O, 25.0; ZnCO_3_, 5.5; MnCl_2_.4H_2_O, 2.5; Cu(OAc)_2_.H_2_O, 0.785; CoCl_3_.6H_2_O, 0.477; CaIO_3_.6H_2_O, 0.295; CrCl_3_.6H_2_O, 0.128; AlCl_3_.6H_2_O, 0.54; Na2SeO_3_, 0.03

Nile tilapia fingerlings (4.5 ± 0.5 g) were obtained from the nursery ponds of Central Laboratory of Aquaculture Research, Abbassa, Abo-Hammad, Egypt, and stocked in a 3-m^3^ fiberglass tank for 2 weeks to be acclimated to indoor laboratory conditions. During this period, the fish were fed with the control diet (30% CP) up to apparent satiety thrice a day. After that, the fish were randomly allocated to fifteen 100-L tanks at a density of 20 fish per tank; tanks were equipped with compressed air through air stones using air pumps. The fish were acclimated once again to tanks’ conditions for other 2 weeks during which fish were fed with the control diet (30% CP) up to apparent satiety thrice a day. After the acclimation period, the tanks were assigned for five treatments in triplicates, and the fish were fed with the experimental diets up to apparent satiety thrice a day at 9:00 h with 4 h intervals for 60 days. Throughout the acclimation period and the experimental period, a half of the water in each tank was siphoned daily along with fish feces and replaced with new well-aerated water from a storage tank. Throughout the acclimation period and the experimental period, the light was kept at 12 h:12 h light-and-dark cycle using fluorescent light tubes.

The water temperature, dissolved oxygen, and pH degree were measured twice daily on site using an automatic probe (Hanna HI-9147), and their ranges were 27.2–29.8°C, 6.1–6.9 mg/L, 7.98–8.39, respectively. The un-ionized ammonia (0.011–0.21 mg/L) was measured using HACH kits (HACH Co., Loveland, CO, USA). All these parameters were within the optimal ranges for fish culture (Boyd and Tucker 2012).

At the end of the experiment, fish were collected from each tank, counted, and group-weighed. Fish growth parameters and feed utilization indices were calculated using the following equations:

$$\mathrm{Weight}\ \mathrm{gain}\ \left(\mathrm{WG}\right)\%=100\ \left({W}_2\hbox{--} {W}_1\right)/{W}_1;$$

$$\mathrm{Specific}\ \mathrm{growth}\ \mathrm{rate}\ \left(\mathrm{SGR};\%/\mathrm{day}\right)=100\ \left[\mathrm{Ln}\ {W}_2\ \left(\mathrm{g}\right)\hbox{--} \mathrm{Ln}\ {W}_1\ \left(\mathrm{g}\right)\right]/T;$$

where *W*_2_ is the final weight (g), *W*_1_ is the initial weight (g), and *T* is the trial period (day).

Feed conversion ratio (FCR) = feed intake/WG; fish survival (%) = 100 (fish number at the end of the experiment/fish number at the start of the experiment).

### Tissues and blood sampling

After the feeding experiment, fish in all tanks were starved for 24 h immediately before blood sampling. Five fish from each tank (*n* = 15 fish per treatment) were anesthetized with sodium bicarbonate-buffered tricaine methane sulfonate (30 mg/L, MS222; Sigma-Aldrich, USA). Blood samples were withdrawn from the caudal vessels of the five fish of each tank and pooled together. Afterward, blood samples were divided into two sections; the first section was performed on the same time using sodium citrate as an anticoagulant for determining the hematological parameters. In the second one, the collected blood samples were allowed to coagulate at room temperature in clean dry centrifuge tubes and then centrifuged for 15 min at 5000 × *g* at room temperature to obtain sera, which were stored at −20°C for further biochemical assays. After blood sampling, the fish were dissected, and samples of the liver and mid-intestine were removed and fixed in 10% neutral formalin for histological examinations.

### Hematological parameters

White blood cells (WBCs) and red blood cells (RBCs) were counted by a hemocytometer in accordance with Brown (1980). The levels of hemoglobin (Hb) and hematocrit (Ht) were calculated using the cyanmethemoglobin method defined by van Kampen and Zijlstra (1961) and Brown (1980), respectively. Furthermore, lymphocytes, neutrophils, and monocytes were counted using an Olympus oil-immersion light microscope with ×1000 magnification.

### Biochemical assays

The blood glucose was evaluated using the methods of Trinder (1969). Serum cholesterol and triglycerides were determined using the methods of Allain et al. (1974) and Fossati and Prencipe (1982), respectively. Serum total protein (TP) and albumin (ALB) were determined using the methods described by Henry (1964) and Doumas et al. (1971), respectively. Serum globulin (GLO) was determined by subtracting the value of ALB from the TP value. Serum alanine (ALT) and aspartate aminotransferase (AST) activities were evaluated using commercial kits (Biodiagnostic Co., Giza, Egypt) and in accordance with Reitman and Frankel (1957). Alkaline phosphatase (ALP) activity was determined following Belfield and Goldberg (1971).

### Digestive enzymes activities

The activities of digestive enzymes in fish sera were determined using fish-specific diagnostic reagent kits (Cusabio Biotech Co. Ltd., Wuhan, Hubei, China) following the manufacturer’s guidelines. Serum amylase, lipase, and protease activities were evaluated following the methods of Bernfeld (1955), Shihabi and Bishop (1971), and Ross et al. (2000), respectively.

### Antioxidant and immunity biomarkers

Diagnostic reagent kits (Biodiagnostic Co., Giza, Egypt) were used to measured antioxidant biomarkers. Malondialdehyde (MDA) content as an indicator to lipid peroxidation was determined in accordance with Ohkawa et al. (1979). The activities of superoxide dismutase (SOD), catalase (CAT), and glutathione peroxidase (GPx) were measured following the methods of McCord and Fridovich (1969), Aebi (1984), and Paglia and Valentine (1967), respectively.

Serum lysozyme (LYZ) was evaluated following the turbidimetric method using *Micrococcus luteus* as the target in phosphate buffer (pH = 6.2) according to Ellis (1990). According to Siwicki and Anderson (1993), total immunoglobulin (total Ig) was measured after polyethylene glycol precipitation of total Ig and the subtraction of initial and final TP.

### Hepatic histology and intestinal histomorphometry

Liver and intestinal specimens were fixed in 10% neutral formalin, dehydrated in ethanol, cleared in xylene, embedded in paraffin blocks, and divided into several 5-μm sections. The prepared tissue sections were then stained with hematoxylin and eosin (H&E) to evaluate the histological characteristics (Bancroft and Gamble 2013). Ten microscopic fields of five slides with sections obtained from five different fish in each group were examined.

The intestinal histomorphometry was estimated in 15 intestinal villi per sample in the mid intestinal part. The effects of dietary C-NPs on the intestinal histomorphometry were evaluated as follows: (a) villus height (from tip to base), (b) villus width at the tip, (c) villus width at the crypt/villus junction, and (d) thickness of the tunica muscularis layer. The computerized quantitative analytics were accomplished on the photomicrographs obtained by a digital camera attached to a bright-field microscope (Nikon, Tokyo, Japan) (Abdel-Tawwab et al. 2021).

### Statistical analysis

The data obtained were subjected to one-way analysis of variance to evaluate the effects of dietary supplementation of C-NPs. Differences between means were tested at 5% probability level using Duncan’s test as a post hoc test. The optimum levels of C-NPs for growth indices and digestive enzymes were determined using polynomial regression analysis (Yossa and Verdegem 2015). All the statistical analyses were conducted via SPSS program version 20 (SPSS, Richmond, VA, USA) according to Dytham (2011).

## Results

### Growth indices and digestive enzymes activities

**Fig. 1:**
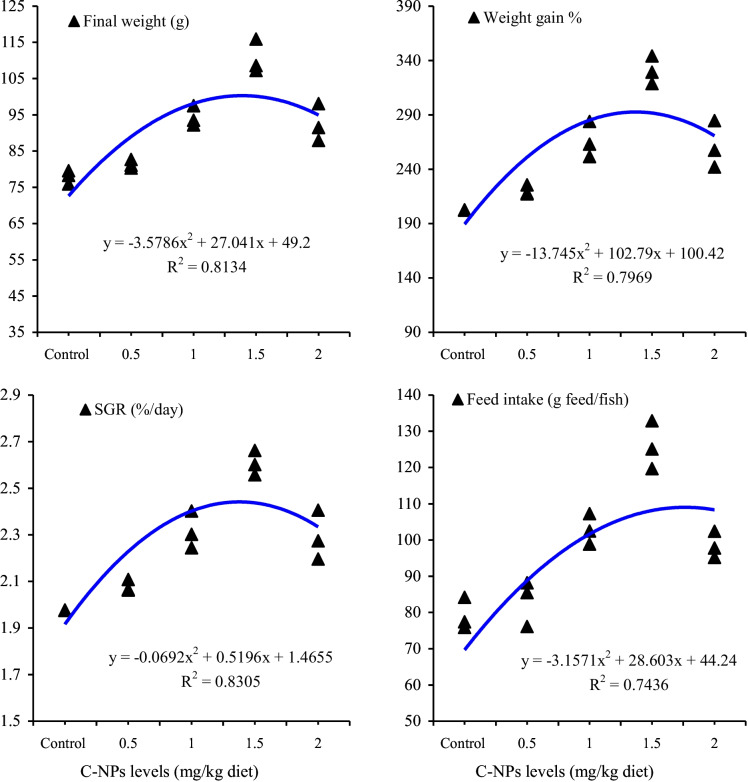
Relationship between various levels of curcumin nanoparticles (C-NPs) and final fish weight (g), SGR (%/day), weight gain %, and feed intake (g feed/fish) of Nile tilapia (*O. niloticus*)

The dietary C-NPs showed positive effects on fish performance in a dose-dependent manner (*P <* 0.05; Table 2). Compared with the control group, the fish fed with C-NPs diets showed significant (*P* < 0.05) enhancements of growth indices, expressed as final weight, WG %, and SGR, and their highest values were observed with treatments of 45 and 60 mg/kg diet. Moreover, fish fed with C-NPs-supplemented diets consumed more feed than those fed with the control diet. The maximum feed intake was observed with fish fed with 45 and 60 mg C-NPs/kg diet (17.2 and 17.6 g feed/fish), whereas those fed with the control diet consumed lowest feed amount (10.0 g feed/fish). FCR values were not significantly (*P >* 0.05) affected by the dietary inclusion of C-NPs in fish diets and ranged from 1.48 to 1.51. The second-order polynomial regression between dietary C-NPs levels and final fish weight (g), SGR (%/day), WG %, or feed intake (g feed/fish) showed that the optimal level of dietary C-NPs for Nile tilapia was between 50 and 55 mg/kg diet (Fig. 1). Throughout the experimental period, the fish in all groups were in good health status, as observed from their general activity, and no mortality (*P >* 0.05) was observed among different treatments (Table 2). These results suggest that dietary C-NPs have no toxic effects on fish health.

**Table 2:** Growth performance and feed utilization of Nile tilapia (*O. niloticus*) fed diets with supplemented with various levels of curcumin nanoparticles (C-NPs) for 60 days (*n* = 3)

	C-NPs levels (mg/kg diet)					*P* value
	0.0 (control)	15	30	45	60	
Initial weight (g)	4.1 ± 0.03	4.2 ± 0.07	4.2 ± 0.07	4.1 ± 0.09	4.3 ± 0.03	0.655
Final weight (g)	18.7 ± 0.38 d	25.2 ± 0.54 c	27.0 ± 0.25 b	29.7 ± 1.28 ab	30.4 ± 1.29 a	<0.001
Weight gain %	354.0 ± 8.45 d	496.1 ± 10.25 c	543.3 ± 14.61 b	618.8 ± 17.17 a	635.1 ± 25.33 a	<0.001
SGR (%/day)	2.52 ± 0.031 d	2.98 ± 0.029 c	3.10 ± 0.038 b	3.29 ± 0.039 a	3.32 ± 0.057 a	<0.001
Feed intake (g feed/fish)	10.0 ± 0.58 c	14.4 ± 0.46 b	15.1 ± 0.73 b	17.2 ± 0.45 a	17.6 ± 0.64 a	0.001
Feed conversion ratio	1.48 ± 0.12	1.49 ± 0.15	1.51 ± 0.03	1.49 ± 0.05	1.49 ± 0.02	1.00
Fish survival (%)	100.0 ± 0.00	100.0 ± 0.00	100.0 ± 0.00	100.0 ± 0.00	100.0 ± 0.00	-

Means having different letters in the same row are significantly different at *P* < 0.05 (Duncan test)

**Fig. 2:**
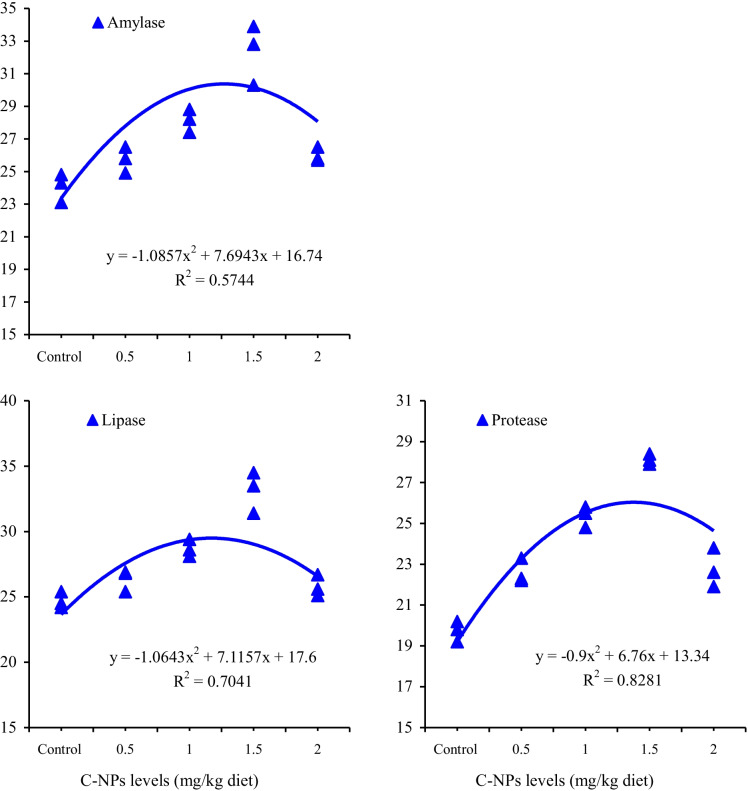
Relationship between various levels of curcumin nanoparticles (C-NPs) and serum amylase, lipase, and protease activities of Nile tilapia (*O. niloticus*)

Feeding the fish with C-NPs diets significantly (*P* < 0.05) enhanced the activities of serum amylase, lipase, and proteases, and their lowest activities were observed in the control group (Table 3). The second-order polynomial regression between activities of these enzymes and dietary levels of C-NPs (Fig. 2) showed that the optimum level of dietary C-NPs suitable for the secretion of digestive enzymes was 45–50 mg/kg diet.

**Table 3:** Activities of serum amylase, lipase, and protease of Nile tilapia (*O. niloticus*) fed with diets supplemented with various levels of curcumin nanoparticles (C-NPs) for 60 days (*n* = 3)

	C-NPs levels (mg/kg diet)					*P* value
	0.0 (control)	15	30	45	60	
Amylase (IU/L)	13.6 ± 0.99 d	16.4 ± 0.83 c	18.8 ± 1.25 b	21.4 ± 1.16 ab	22.7 ± 1.58 a	0.006
Lipase (IU/L)	21.7 ± 1.21 c	25.4 ± 0.99 b	31.5 ± 0.57 a	33.7 ± 1.56 a	34.1 ± 0.72 a	<0.001
Protease (IU/L)	19.6 ± 0.38 d	24.6 ± 0.65 c	26.1 ± 0.67 b	28.7 ± 0.57 a	29.4 ± 1.26 a	<0.001

Means having different letters in the same row are significantly different at *P* < 0.05

### Hematological parameters

Table 4 shows the effects of different levels of dietary C-NPs on the hematological parameters. The WBCs and RBCs counts, Hb concentration, and Ht level of the fish fed with 30–60 mg C-NPs/kg diet were statistically (*P* < 0.05) higher than those of the control group; however, their levels were approximately (*P* > 0.05) similar. Moreover, lymphocytes % and monocytes % significantly (*P* > 0.05) increased; meanwhile neutrophils % significantly (*P* > 0.05) decreased as C-NPs levels in diets increased (Table 4).

**Table 4:** Hematological parameters of Nile tilapia (*O. niloticus*) fed diets supplemented with various levels of curcumin nanoparticles (C-NPs) for 60 days (*n* = 3)

	C-NPs levels (mg/kg diet)					*P* value
	0.0 (control)	15	30	45	60	
RBCs (1x10^6^ uL)	3.12 ± 0.047 c	3.41 ± 0.084 b	3.73 ± 0.094 ab	3.84 ± 0.182 a	3.94 ± 0.201 a	0.021
Hemoglobin (mg/dL)	7.2 ± 0.48 b	9.3 ± 0.26 a	9.7 ± 0.13 a	10.3 ± 0.64 a	10.7 ± 1.29 a	0.015
Hematocrit (%)	38.2 ± 1.14 c	45.4 ± 1.75 b	51.0 ± 1.96 a	51.7 ± 2.61 a	51.9 ± 4.21 a	0.029
WBCs (×10^3^ uL)	18.2 ± 0.98 c	22.4 ± 0.34 b	25.3 ± 3.14 ab	26.2 ± 3.39 a	27.9 ± 2.62 a	0.023
Lymphocytes (%)	65.4 ± 1.99 a	52.3 ± 2.89 b	51.1 ± 2.61 b	45.9 ± 1.39 c	43.9 ± 1.86 c	<0.001
Neutrophils (%)	22.6 ± 1.05 c	29.3 ± 1.44 b	30.3 ± 0.69 b	32.9 ± 1.82 ab	35.9 ± 2.15 a	0.001
Monocytes (%)	11.3 ± 0.61 c	17.6 ± 1.21 b	19.2 ± 0.83 a	20.4 ± 1.22 a	21.1 ± 0.36 a	<0.001

*RBCs* Red blood cells; *WBCs* white blood cells

Means having different letters in the same row are significantly different at *P* < 0.05

### Biochemical parameters

The levels of blood glucose, total cholesterol, and triglyceride of C-NPs-fed fish were significantly (*P* < 0.05) lower than the control group, and their lowest levels were recorded in the fish fed with 30–60 mg C-NPs/kg diet (Table 5). On the other hand, TP, ALB, and GLO in serum of C-NPs-fed Nile tilapia were significantly (*P* < 0.05) higher than those of the control group, and their highest levels were recorded in the fish fed with 45 and 60 mg C-NPs/kg diet with no significant deference between them (Table 5). Serum AST, ALT, and ALP activities were not significantly affected by the dietary C-NPs (Table 5). These results suggest that the fish were not stressed.

**Table 5:** Changes in hemato-biochemical parameters of Nile tilapia (*O. niloticus*) fed with diets supplemented with various levels of curcumin nanoparticles (C-NPs) for 60 days (*n* = 3)

	C-NPs levels (mg/kg diet)					*P* value
	0.0 (control)	15	30	45	60	
Glucose (mg/dL)	113.9 ± 4.58 a	91.3 ± 3.95 b	89.6 ± 5.65 b	85.3 ± 5.59 bc	81.9 ± 4.96 c	0.001
Total cholesterol (mg/dL)	192.5 ± 2.41 a	195.4 ± 2.11 a	180.0 ± 1.82 b	174.2 ± 5.61 c	174.3 ± 2.58 c	0.033
Triglyceride (mg/dL)	152.2 ± 5.86 a	147.1 ± 8.67 a	143.9 ± 8.83 a	113.0 ± 3.88 b	115.7 ± 9.49 b	0.008
Total protein (g/dL)	4.06 ± 0.042 d	4.90 ± 0.211 c	5.25 ± 0.181 bc	5.57 ± 0.285 ab	5.95 ± 0.086 a	<0.001
Albumin (g/dL)	2.68 ± 0.352 c	3.23 ± 0.035 b	3.53 ± 0.131 ab	3.59 ± 0.196 a	3.98 ± 0.074 a	0.009
Globulin (g/dL)	1.38 ± 0.316 c	1.67 ± 0.197 b	1.72 ± 0.195 b	1.98 ± 0.263 a	1.97 ± 0.252 a	0.035
AST (IU/L)	12.4 ± 0.61	12.3 ± 0.54	12.2 ± 0.25	12.2 ± 0.24	12.3 ± 0.29	0.983
ALT (IU/L)	41.9 ± 0.81	42.1 ± 0.88	42.8 ± 1.19	42.4 ± 0.94	42.5 ± 0.67	0.966
ALP (IU/L)	30.7 ± 0.29	31.0 ± 0.27	31.6 ± 0.97	30.4 ± 0.53	31.0 ± 0.32	0.841

*AST* aspartate aminotransferase; *ALT* alanine aminotransferase; *ALP* alkaline phosphatase

Means having different letters in the same row are significantly different at *P* < 0.05

### Antioxidant and immunity biomarkers

Table 6 shows the effects of different levels of C-NPs in diets on the antioxidant status and immunity response of Nile tilapia. At the end of the feeding trial, the C-NPs-fed fish showed significantly (*P* < 0.05) lower MDA levels than the control group, and the lowest MDA levels were significantly (*P* < 0.05) observed in fish fed with 45–60 mg/kg diet with no significant difference between them. On the other hand, the fish fed with C-NPs diets for 60 days had significantly (*P* < 0.05) higher SOD, CAT, and GPx activities than those fed with the control diet, and their highest (*P* < 0.05) activities were recorded at 45 and 60 mg C-NPs/kg diet with no significant difference between them (*P* > 0.05). Furthermore, feeding the fish with C-NP-containing diets significantly (*P* < 0.05) stimulated the LYZ activity and total Ig level over than the control group (Table 6). These immunological parameters of fish fed with 45 and 60 mg C-NPs/kg diet were the highest with no significant (*P* > 0.05) difference between them.

**Table 6:** Serum antioxidants and immunological responses of Nile tilapia (*O. niloticus*) fed with diets supplemented with various levels of curcumin nanoparticles (C-NPs) for 60 days (*n* = 3)

	C-NPs levels (mg/kg diet)					*P* value
	0.0 (control)	15	30	45	60	
MDA (nmol/dL)	2.18 ± 0.115 a	1.91 ± 0.106 ab	1.75 ± 0.075 bc	1.59 ± 0.71 cd	1.51 ± 0.112 d	0.004
SOD (IU/L)	13.4 ± 0.41 d	16.3 ± 1.42 c	18.4 ± 0.91 b	21.3 ± 0.94 a	22.6 ± 1.21 a	<0.001
CAT (IU/L)	10.1 ± 0.29 d	12.5 ± 0.61 c	14.4 ± 0.69 b	15.3 ± 0.73 ab	15.7 ± 0.49 a	<0.001
GPx (IU/L)	19.3 ± 0.36 c	22.7 ± 0.51 b	25.4 ± 0.35 a	26.5 ± 0.55 a	26.7 ± 0.61 a	<0.001
LYZ (μg/mL)	1.37 ± 0.024 d	1.78 ± 0.285 c	2.18 ± 0.125 b	2.47 ± 0.208 ab	2.64 ± 0.176 a	<0.001
Total Ig (mg/mL)	18.9 ± 0.64 d	22.4 ± 0.49 c	25.3 ± 0.57 b	28.6 ± 1.28 a	29.2 ± 0.99 a	<0.001

*MDA* Malondialdehyde; *SOD* superoxide dismutase; *CAT* catalase; *GPx* glutathione peroxidase; *LYZ* lysozyme; *total Ig* total immunoglobulin

Means having different letters in the same row are significantly different at *P* < 0.05

### Liver histology and intestinal histomorphometry

**Fig. 3:**
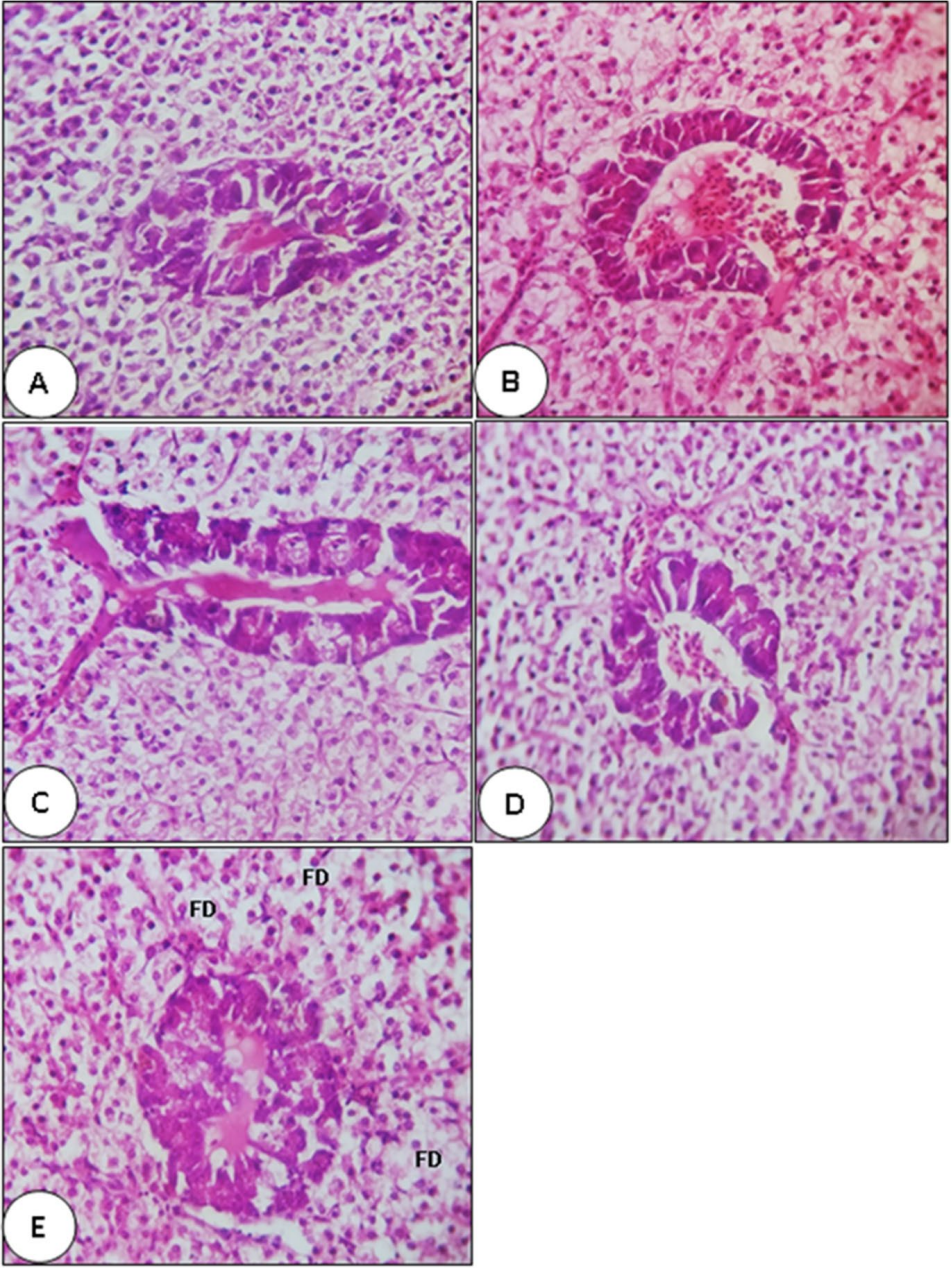
Photomicrograph (×400, H&E stain) of hepatopancreas of Nile tilapia (*O. niloticus*) fed with different levels of curcumin nanoparticles (C-NPs) for 60 days: 0.0 (the control) (**A**), 15 (**B**), 30 (**C**), 45 (**D**), and 60 mg/kg diet (**E**). Plate A shows the normal structure of hepatopancreas cells in the control group. Plate B shows the normal structure with slight improvement of hepatopancreas cells. Plate C shows moderate improvement in the hepatopancreatic structure. Plate D shows a normal structure with marked improvement in hepatopancreatic cells structure. Plate E shows a marked improvement in hepatopancreatic cells structure with some fat deposition (FD)

**Fig. 4:**
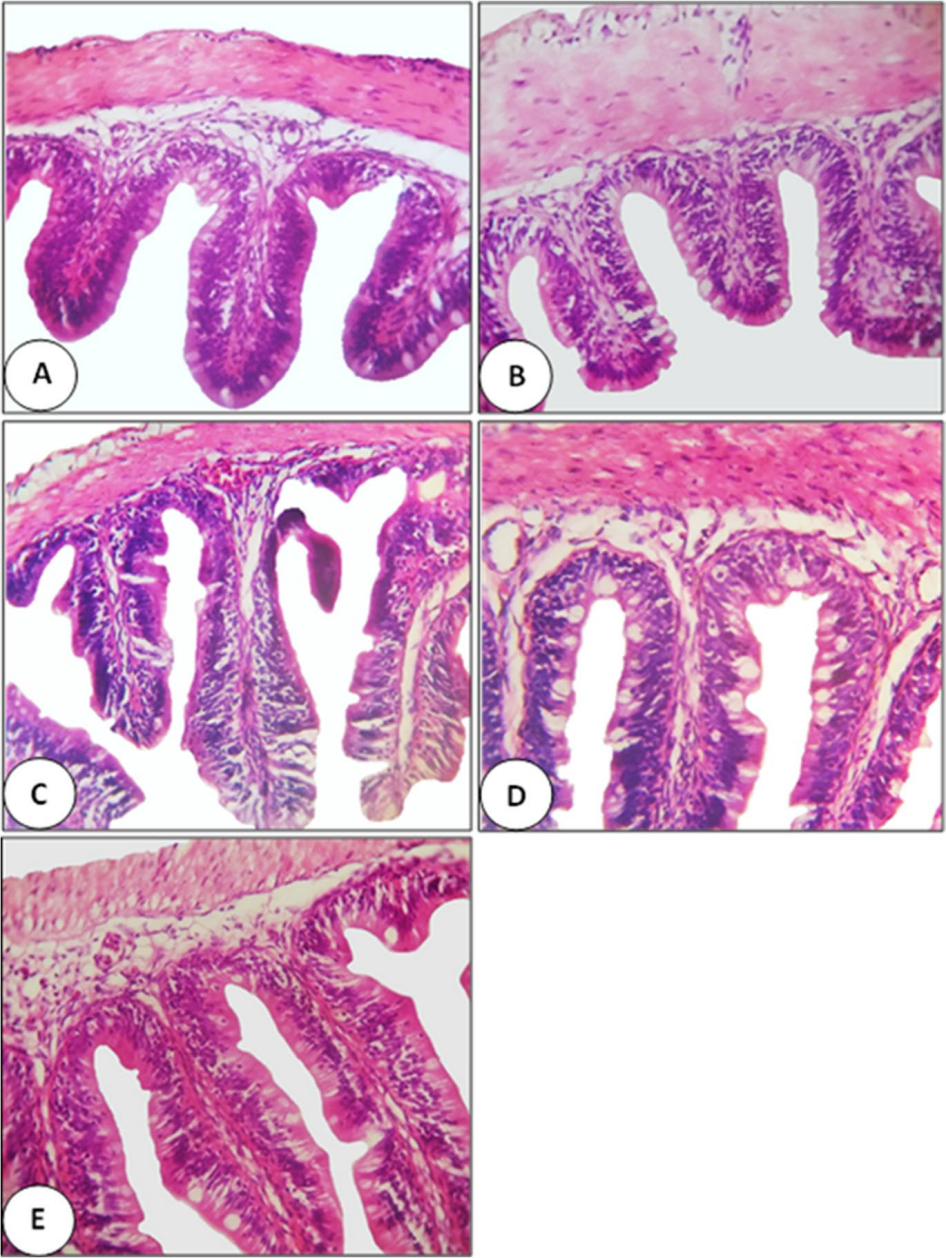
Photomicrograph (×400, H&E stain) of the mid-intestine of Nile tilapia (*O. niloticus*) fed with different levels of curcumin nanoparticles (C-NPs): 0.0 (the control) (**A**), 15 (**B**), 30 (**C**), 45 (**D**), and 60 mg/kg diet (**E**). Plate A shows normal histological structures of intestinal villi, crypt, and tunica muscularis with normal epithelium, goblet cells, and lamina propria. Plate B shows a normal structure with slight improvement in the structure of several villi cells. Plate C shows moderate improvement in the intestinal structure. Plate D shows a normal structure with marked improvement in the intestinal structure. Plate E shows a marked improvement in the intestinal structure

The liver of the control and C-NPs-fed fish in the present study exhibited a normal structure, and no pathological abnormalities were observed. The liver of the control fish consisted of compactly arranged hepatocytes, several blood capillaries, and hepatopancreas (Fig. 3). Marked improvements in hepatopancreatic structure were observed with the increase in levels of dietary C-NPs up to 45 mg/kg diet after which (60 mg C-NPs/kg diet) fat drops were found (Fig. 3). On the other hand, the mucosa and submucosa of the mid-intestine of Nile tilapia in the control group and in fish group fed with C-NPs diets had a normal histomorphology (Fig. 4). Significant increases in the density, length, and branching of intestinal villi were illustrated in all C-NP-fed fish groups compared with the control diet group. The intra-epithelial lymphocytes showed noticeable increases in the C-NP-supplemented groups alongside the presence of lymphocytic aggregations in the propria submucosa of fish fed with 45 and 60 mg C-NPs/kg diet (Fig. 4). Moreover, significant improvements (*P* <0.05) in villus height/width (at the tip and crypt/villus junction) and absorption area were observed in fish groups fed with C-NPs diets in a dose-dependent manner (Table 7). Interestingly, the muscular thickness of the mid intestine of Nile tilapia significantly increased as the levels of C-NPs increased up to 30–60 mg/kg diet with no significant difference among them (Table 7).

**Table 7:** Intestinal morphology of Nile tilapia (*O. niloticus*) fed with diets supplemented with various levels of curcumin nanoparticles (C-NPs) for 60 days (*n* = 3)

	C-NPs levels (mg/kg diet)					*P* value
	0.0 (control)	15	30	45	60	
Villus length (μm)	131.0 ± 8.59 d	152.8 ± 9.26 c	184.6 ± 12.16 b	189.3 ± 11.53 ab	213.6 ± 10.86 a	0.001
Villus width (μm)	65.5 ± 4.19 c	72.5 ± 2.75 b	75.8 ± 2.13 b	82.0 ± 3.17 a	83.5 ± 3.83 a	<0.001
Muscular thickness (μm)	21.3 ± 1.40 c	25.7 ± 1.99 b	29.0 ± 1.32 a	31.9 ± 1.38 a	32.6 ± 1.33 a	<0.001
Absorption area (μm^2^)	8580.5 ± 601.2 c	11078.0 ± 1026.8 b	13992.7 ± 891.9 b	15522.6 ± 1296.2 ab	17835.6 ± 861.1 a	<0.001

Means having different letters in the same row are significantly different at *P* < 0.05

## Discussion

### Growth performance and digestive enzymes

The present study revealed that dietary C-NPs significantly enhanced the growth performance of Nile tilapia with optimum levels of 45–55 mg/kg diet. These results may be attributed to the significant enhancements of intestinal morphometry, leading to the enlarged gut surface area with increasing the villi width/height and subsequently improved the nutrient absorption. Additionally, dietary C-NPs may increase the feed palatability, leading to increased feed intake, as observed in the present study. The same growth stimulating effects were previously reported by dietary supplementation of curcumin in crucian carp (Jiang et al. 2016), rainbow trout (Akdemir et al. 2017; Enis Yonar et al. 2019), grass carp, *Ctenopharyngodon idella* (Ming et al. 2020), common carp, *Cyprinus carpio* (Abdel-Tawwab and Abbass 2017; Giri et al., 2019), Nile tilapia, *O*. *niloticus* (Priyadarsini 2014; Mahmoud et al. 2017; Mohamed et al. 2020), and Mozambique tilapia, *Oreochromis mossambicus* (Sruthi et al. 2018). Moghadam et al. (2021) fed Pacific white shrimp (*Penaeus vannamei*) with diets containing 0.0, 75, 150, and 300 mg of curcumin nanomicelles (NMC) per kg diet for 63 days. They found that diet containing 150 mg NMC/kg diet showed the highest performance of Pacific white shrimp.

Curcumin is a polyphenolic compound extracted from turmeric and may act as a growth-stimulator for beneficial gut bacteria (acting in a prebiotic-like manner), which can inhibit the growth of pathogenic bacteria (Gessner et al. 2017). The enhancements in fish performance may also be attributed to the digestion-enhancing properties of curcumin. In this regard, the incorporation of C-NPs in diets of Nile tilapia stimulated the activities of serum amylase, lipase, and protease. The activities of digestive enzymes in fish serum or gut were higher in fish fed with curcumin when compared with the control (Midhun et al. 2016; Jiang et al. 2016; Sruthi et al. 2018). In similar studies, increasing the digestive enzymes activities resulted in improving the growth performance of Nile tilapia fed with graded levels of cinnamon nanoparticles (Abdel-Tawwab et al. 2018a) and cinnamaldehyde nanoemulsion (Abd El-Hamid et al. 2021).

### Hemato-biochemical assays

Hemato-biochemical parameters are important tools commonly used in evaluating fish health status, nutritional status as well as the adaptation capability of fish to the external environment (Abdel-Tawwab 2016; Adeshina et al. 2019). The results revealed that the RBCs count, Hb concentration, and Ht level in C-NPs-fed fish were significantly higher than those of the control group. These results showed that the dietary C-NPs had positive effects on the hematological parameters of Nile tilapia without anemia symptoms. These findings can be linked to the promotion of erythropoiesis and hemosynthesis, indicating the enhancement of fish health. These results agree with previous researches, which reported that dietary inclusion of curcumin enhances the hematological parameters, including WBCs counts, in different fish species (Mişe Yonar et al. 2013; Priyadarsini, 2014; El-Barbary 2018; Enis Yonar et al. 2019; Mohamed et al. 2020).

Lymphocytes and monocytes percentages significantly (*P* > 0.05) increased; meanwhile neutrophils percentage significantly (*P* > 0.05) decreased as C-NPs levels in diets increased (Table 4). Monocytes are referred to as macrophages and are responsible for the processing and presenting antigens to lymphocytes, linking the innate and adaptive immune system (Zachary et al. 2018; Pereira et al. 2020). The higher presence of circulating monocytes and lymphocytes in C-NPs-fed fish is associated with the improved immunomodulatory properties. Similar results were observed by Sahu et al. (2008) in *Labeo rohita* fed a diet supplemented with turmeric, *C. longa.* Pereira et al. (2020) found that feeding Nile tilapia on diets with turmeric hydrolate resulted in a significant increase of monocytes and lymphocytes in relation to the control group.

AST and ALT are critical aminotransferases in the liver, reflecting the liver status (Murray et al. 2003). The C-NPs inclusion in diets for Nile tilapia did not significant (*P >* 0.05) affect serum AST, ALT, and ALP activities. These results suggest that fish in the present study were unstressed. Abdelkhalek et al. (2021) demonstrated significant decreases in serum AST and ALT activity in fish fed on curcumin-supplemented diets. Moghadam et al. (2021) reported that the levels of ALT and AST decreased in *P*. *vannamei* fed with different dosages of NMC additives compared with the control especially at the treatment of 150 mg NMC/kg diet. In other studies used phytogenic materials, Abd El-Hamid et al. (2021) found no significant (*P* > 0.05) differences in serum AST and ALT levels in fish fed diets with different levels of cinnamaldehyde nanoemulsion. Abdel-Latif et al. (2020) reported that activities of serum ALT, AST, and ALP levels were not significantly (*P* < 0.05) differed in fish fed with different levels of oregano essential oil as compared to the control group.

Significant (*P* < 0.05) reductions in the levels of glucose, total cholesterol, and triglycerides were found in Nile tilapia fed with C-NPs diets as compared with the control group. In this regard, Sruthi et al. (2018) reported that the blood glucose levels significantly decreased in fish fed with curcumin-enriched diets compared with the control. They suggested that curcumin promoted the glucose uptake and glycogenesis, reducing the blood glucose level. The current study also suggests that curcumin may play a modulatory role in the activity of enzymes involved in lipid homeostasis, reducing the total cholesterol and triglyceride levels. These results may be because curcumin limits the cholesterol biosynthesis and lowers the plasma and hepatic cholesterol concentrations (Shin et al. 2011). Additionally, curcumin modulates hepatic gene expression, inhibits cholesterol biosynthesis via down-regulation of major lipogenic factors (Kang and Chen 2009; Shin et al. 2011; Zhao et al. 2011), stimulates bile acid secretion, and enhances the clearance of cholesterol as bile (Kim and Kim 2010; Prakash and Srinivasan 2011).

### The antioxidant activity and lipid peroxidation

The antioxidant system is greatly related to health and immunity, and several studies have described a positive relation between the antioxidant activity and immune response of fish and shellfish. SOD, CAT, and GPx as main endogenous enzymes of this system, preserve the cells from oxidative stress and catalyse the reactive oxygen species (ROS) into less reactive forms (Livingstone 2001; Abdel-Tawwab and Wafeek 2017; Hoseinifar et al. 2021). Additionally, MDA is the product of lipid peroxidation and indicates the oxidative damages to the lipids. The findings of this study indicate that feeding Nile tilapia with C-NPs significantly increased SOD, CAT, and GPx activities along with significant decreases in MDA levels. These results indicate to the antioxidant activity of C-NPs. It is known that curcumin could scavenge free radicals and stimulating antioxidant parameters (Manju et al. 2012; Xu et al. 2018). Another mechanism for antioxidant properties of curcumin is the transcription induction of antioxidant enzymes by activating the nuclear factor erythroid 2 (Nrf2) signaling pathway, which is involved in the free radical scavenging (Kwak et al. 2004). On the other hand, the antioxidant properties of curcumin could be linked to its high content of polyphenols, which act as hydrogen or electron donors and has the capability to stabilize unpaired electrons and terminate Fenton reactions (Bishayee et al. 2011). Moreover, polyphenolic compounds are nitrosation reaction inhibitors and can prevent oxidative damage via reactive oxygen species scavenging and enhancing the SOD and GPx activities (Moskaug et al. 2005). In a similar study, Moghadam et al. (2021) found significant increases of SOD and CAT activities coupled with lower MDA levels in *P*. *vannamei* fed with different dosages of NMC additives especially at the treatment of 150 mg NMC/kg diet. Previous studies demonstrated that dietary supplementation of curcumin significantly enhanced the antioxidant activity of Nile tilapia (Mahmoud et al. 2017), crucian carp (Jiang et al. 2016), common carp (Yonar 2018), and rainbow trout (Enis Yonar et al. 2019).

### Immunological responses

Serum TP, which is made up of ALB and GLO, is often measured as an indicator associated with the health and immune status in aquatic animals and their increases displays a more robust innate immune status. LYZ is the first-line barrier of the defense system, and it causes lysis of bacteria and activation of the complement system and phagocytes (Magnadottir 2010). Serum immunoglobulins are a major component of the vertebrate humoral immune system, and they play a vital role in immune processes, such as phagocytosis, opsonization, and neutralization of pathogenic bacteria, viruses, and toxins in the host body (Cuesta et al. 2004; Magnadottir 2010). The present results indicated noticeable increases in TP, ALB, GLO, LYZ, and total Ig levels in fish fed with C-NPs, and this finding are associated with the induction of the humoral immunity of Nile tilapia. In a similar study, Moghadam et al. (2021) reported that feeding *P*. *vannamei* on NMC-enriched diets showed higher levels of LYZ, TP, and ALB than the control group. Positive effects of curcumin on the innate immunity of different fish species were previously found (Mahmoud et al. 2017; Enis Yonar et al. 2019). Jagetia and Aggarwal (2007) observed that dietary curcumin enhanced the fish immunity by activating macrophages and neutrophils. Antony et al. (1999) also stated that curcumin increases the production of cytokines, which play key roles in regulating the immunity response.

### Liver histology and intestinal histomorphometry

The hepatopancreatic and intestinal tissues in all experimental groups in the present study showed normal histological structures with no evident inflammatory responses, suggesting the absence of toxic influences of C-NPs on fish tissues. Marked improvements in hepatopancreatic structure were observed with the increase in levels of dietary C-NPs up to 45 mg/kg diet after which (60 mg C-NPs/kg diet) fat drops were found. In this regard, Manju et al. (2012) found no histological changes in the liver of *Anabas testudineus* fed with 5 and 10 g/kg diet of curcumin. Conversely, in the study of El-Barbary (2018), Nile tilapia fed with 20 g per kg diet of curcumin exhibited the degeneration of vacuoles in hepatocytes and large accumulation of hemosiderin around blood vessels. Therefore, the effects of curcumin on fish are dependent on its levels, feeding period, fish species, and fish size.

The intestine is the main site for nutrient absorption in fish, and it is where nutrients are transported into and out of the intestinal enterocytes by specific transporters located at the brush-border and basolateral membranes (Broer 2008; Nicholson et al. 2012; Abdel-Tawwab et al. 2018b; Adeshina et al. 2019; Abdel-Latif et al. 2020). In regard to the intestinal histomorphometry, the dietary supplementation of C-NPs to Nile tilapia significantly enhanced the villi length/width and absorption area in the mid-intestine in a dose-dependent manner. However, long villi are usually associated with outstanding gut health, great nutrient, and absorption efficiency, leading to improved performance (Sklan et al. 2004; Trushenski 2015; Huerta-Aguirre et al. 2019). These findings suggest that dietary C-NPs may reduce gut inflammation and subsequently exert positive effects on gut health, nutrient absorption, and thereby the growth of Nile tilapia. Giannenas et al. (2010) reported that the administration of phenolic compounds, such as curcumin, may reduce gut inflammation, leading to improvement of nutrient digestibility and metabolism. Namagirilakshmi et al. (2010) and Kosti et al. (2018) reported that dietary turmeric significantly increased the intestinal villi length/width than the control diet. A positive correlation between villus height/width and fish growth was previously reported (Abdel-Tawwab et al. 2018b; Adeshina, et al. 2019). The enhancement of gut morphology increases the nutrient uptake, which improves the feed utilization and growth performance of numerous fish species (Abdel-Tawwab et al. 2018b; Adeshina et al. 2019; Abdel-Latif et al. 2020; Abdel-Tawwab et al. 2021).

## Conclusions

The current study showed that dietary C-NPs positively enhanced the growth performance, digestive enzymes, hemato-biochemical indices, antioxidant status, and humoral immunity of Nile tilapia. These results indicate that C-NPs can be considered as a beneficial dietary supplement for Nile tilapia with optimum inclusion levels of 45–55 mg/kg diet.

## Data Availability

Data of the present article are available under request.
